# BMIgap: a new tool to quantify transdiagnostic brain signatures of current and future weight

**DOI:** 10.21203/rs.3.rs-5259910/v1

**Published:** 2024-12-11

**Authors:** Nikolaos Koutsouleris, Adyasha Tejaswi Khuntia, David Popovic, Elif Sarisik, Madalina O. Buciuman, Mads L. Pedersen, Lars T. Westlye, Ole Andreassen, Andreas Meyer-Lindenberg, Joseph Kambeitz, Raimo Salokangas, Jarmo Hietala, Alessandro Bertolino, Stefan Borgwardt, Paolo Brambilla, Rachel Upthegrove, Stephen Wood, Rebekka Lencer, Eva Meisenzahl, Peter Falkai, Emanuel Schwarz, Ariane Wiegand

**Affiliations:** Ludwig-Maximilians-University; Ludwig-Maximilian-University; Max-Planck-Institute of Psychiatry; Ludwig-Maximilians-University; Ludwig-Maximilians-University; University of Oslo; University of Oslo; Oslo University Hospital & Institute of Clinical Medicine, University of Oslo; Central Institute of Mental Health, Mannheim; Faculty of Medicine and University Hospital University of Cologne Cologne; Department of Psychiatry, University of Turku; Department of Psychiatry, University of Turku and Turku University Hospital, Finland; Department of Translational Biomedicine and Neuroscience, University of Bari “Aldo Moro”, Bari, Italy; University of Lübeck; University of Milan; University of Birmingham; University of Melbourne; Department of Psychiatry and Psychotherapy and Center for Brain, Behaviour and Metabolism, University of Lübeck; Kliniken der Heinrich-Heine-Universität; Ludwig Maximilians University Munich; Central Institute of Mental Health; Ludwig-Maximilian-University

**Keywords:** BMI, machine learning, grey matter volume, schizophrenia, major depressive disorder

## Abstract

Understanding the neurobiological underpinnings of weight gain could reduce excess mortality and improve long-term trajectories of psychiatric disorders. We used support-vector machines and whole-brain voxel-wise grey matter volume to generate and validate a BMI predictor in healthy individuals (N = 1504) and applied it to individuals with schizophrenia (SCZ,N = 146), clinical high-risk states for psychosis (CHR,N = 213) and recent-onset depression (ROD,N = 200). We computed BMIgap (BMI_predicted_-BMI_measured_), interrogated its brain-level overlaps with SCZ and explored whether BMIgap predicted weight gain at 1- and 2-year follow-up. SCZ (BMIgap = 1.05kg/m^2^) and CHR individuals (BMIgap = 0.51 kg/m^2^) showed increased and ROD individuals (BMIgap=−0.82 kg/m^2^) decreased BMIgap. Shared brain patterns of BMI and SCZ were linked to illness duration, disease onset, and hospitalization frequency. Higher BMIgap predicted future weight gain, particularly in younger ROD individuals, and at 2-year follow-up. Therefore, we propose BMIgap as a potential brain-derived measure to stratify at-risk individuals and deliver tailored interventions for better metabolic risk control.

## Introduction

Globally 26% of adults are overweight (BMI of 25–30 kg/m^2^) with an additional 13% classified as obese (BMI ≥ 30 kg/m^2^) (1), highlighting the pandemic nature of obesity (2, 3). Given its strong associations with metabolic diseases, including cardiovascular disease and type 2 diabetes, obesity stands as a major risk factor for somatic disorders (4). Importantly, obesity frequently parallels psychiatric disorders such as schizophrenia (SCZ), major depressive disorder (MDD), bipolar, personality and anxiety disorders (5, 6). Psychiatric patients have a two-to-three-fold higher incidence of obesity and metabolic diseases compared to the general population, significantly contributing to their excess mortality (7, 8). Secondary factors such as smoking, alcohol use, sedentary lifestyle, and commonly prescribed medications also significantly impact body weight and metabolic syndromes in psychiatric patients (9, 10). Particularly atypical antipsychotics and antidepressants have been implicated in weight gain (11, 12). Furthermore, individuals exhibiting negative symptoms–characterized by affective flattening, anhedonia and avolition–are at an increased risk for weight gain (13). In turn, weight gain and obesity can negatively impact an affected individual’s quality of life and self-esteem, thereby further exacerbating already pre-existing mental health issues and ultimately leading to a vicious circle between psychiatric symptoms, secondary disease effects and weight gain (14, 15).

In healthy populations, neuroimaging studies have identified associations between higher BMI and reduced grey matter volume (GMV) in the prefrontal, temporal, parietal and occipital cortices, the cerebellum, insula, thalamus, and amygdala (16–18). These associations might be related to lowered inhibitory control activity for food-related stimuli and responses resulting from a disbalance of brain activity in cognitive control regions and reward-related regions (17–19). These findings suggest a plausible relationship between cognitive control deficits and overeating (19) potentially resulting in higher caloric intake and obesity (17). Furthermore, in patients with SCZ, higher BMI was found to be associated with reduced GMV in the prefrontal cortex (PFC), specifically the orbitofrontal cortex (OFC), and the hippocampus (20). In patients with MDD, higher BMI was correlated with reduced GMV in the medial prefrontal regions, particularly in areas involved in impulse control and emotion regulation (21).

However, a comprehensive understanding of the neurobiological underpinnings of obesity, particularly in in psychiatric disorders, is lacking as are methods to identify those at risk for weight gain (22, 23). Therefore, personalized tools are needed to capture these interactions and enable targeted interventions–such as exercise, psychotherapy, medications, or brain stimulation–to prevent weight gain, improve treatment adherence, and reduce excess mortality (24, 25).

To better understand the neurobiological underpinnings of BMI and weight gain in early stages of affective and psychotic disorders, we developed an individualized BMI prediction model based on whole-brain GMV in healthy control (HC) individuals (HC_discovery_) and validated it in a HC sample (HC_validation_). We then applied this BMI predictor to individuals suffering from schizophrenia (SCZ), recent-onset depression (ROD), and clinical high-risk states for psychosis (CHR). We introduce BMIgap (Body Mass Index gap estimation) as an individualized brain-based metric, which calculates the difference between brain-estimated and measured BMI (BMI_predicted_-BMI_measured_). Next, we correlated BMIgap with future weight changes at 1- and 2-years follow-up at the group level and used it along with additional clinical factors to predict future weight gain at the individual level. Finally, we explored the phenotypic association between BMIgap, SCZ expression (i.e. neuroanatomical similarity for SCZ), and clinical features using sparse partial least squares (SPLS). We hypothesized that (i) accurate models for individualized BMI prediction can be derived from structural brain imaging using supervised machine learning, (ii) interactions between BMI-predictive and disease-specific brain signatures result in systematic brain deviations that are captured by BMIgap, (iii) BMIgap is associated with key measures of disease severity, including age of onset, illness duration or hospitalization frequency, and (iv) BMIgap can serve as a personalized brain-based tool to assess future weight gain and identify at- risk individuals in early disease stages.

## Materials and methods

### Participants

The study followed the Transparent Reporting of a Multivariable Prediction Model for Individual Prognosis or Diagnosis (TRIPOD) reporting guidelines (https://www.equator-network.org/reporting-guidelines/tripod-statement/). We included T1-weighted MRI scans of 1504 HC individuals (HC_discovery_=770; HC_validation_=734) from four independent datasets covering 14 sites: Information eXtraction from Images (IXI; https://brain-development.org/ixi-dataset/), Personalized Prognostic Tools for Early Psychosis Management (PRONIA; www.pronia.eu), Norwegian Centre for Mental Disorders Research (NORMENT; https://www.med.uio.no/norment/ (26)) and the Munich Brain Imaging Database (MUC, (27)) (Supplementary Methods). Moreover, three patient populations were included: SCZ individuals (N = 146) from the MUC cohort and CHR (N = 213) and ROD individuals (N = 200) from the PRONIA study (Table 1, Table S2). All studies were approved by their local ethics committee and adhered to the ethical standards as outlined in the Declaration of Helsinki (28).

We included HC individuals aged 15–75 years with a BMI of 18.5–35 kg/m^2^ without current or previous psychiatric disorders, in the discovery model by following these steps: (i) to avoid an underrepresentation of the tails of the BMI distribution and to maximize model generalizability throughout the investigated BMI range, we sampled 770 HC individuals into 33 BMI bins (0.5 BMI per bin) to approximate a uniform BMI distribution (Figure S1a); (ii) age distribution of individuals was matched within the BMI bins to mitigate the natural correlation between age and BMI (29) (Figure S1 c). These 770 HC individuals constituted the HC_discovery_ sample, and the remaining 734 HC individuals constituted the HC_validation_ sample. The clinical population consisted of individuals with SCZ (N = 146), CHR (N = 213) and ROD (N = 200) (Figures S1-S2, Supplementary Methods).

### MRI data acquisition and pre-processing

The Participants in the IXI and PRONIA studies underwent MRI scanning at 1.5T or 3T, while NORMENT and MUC participants were examined at 1.5T MRI scanners (Supplementary Methods, Table S1). To facilitate between-study comparability, we applied the VBM8 pre-processing pipeline described by Opel et al (18) to produce normalized, modulated GMV tissue maps (Supplementary Methods). For computational efficiency and noise reduction, GMV images were resliced to a 3×3×3 mm^3^ isotropic voxel resolution.

## Machine learning analysis

The open-source machine learning software NeuroMiner (v1.1; https://github.com/neurominer-git/NeuroMiner_1.1) was used for training and application of all supervised machine learning models. To prevent information leakage between train and test data and enhance model generalizability, we implemented a repeated nested cross-validation (CV) cycle with five folds and permutations each at the inner and outer CV cycles. Thus, the process of model optimization was completely insulated from the process of model validation (Supplementary Methods).

Data pre-processing included Gaussian smoothing of the neuroimaging data, partial correlation analysis to remove effects of chronological age from the GMV data, global mean offset correction to remove site effects, i.e. MR scanner effects, principal component analysis for dimensionality reduction, and voxel-wise scaling between 0 and 1 (Supplementary Methods). We used p-support vector machine regression with a linear kernel to predict BMI based on whole-brain voxel-wise GMV (71276 features) using the mean absolute error (MAE) as the optimization criterion. The statistical significance of the model was evaluated using 1000 permutations of the BMI label (α = 0.05).

Predictive brain patterns were visualized at the voxel level combining the grand means of the cross-validation ratio (CVR) (30) and sign-based consistency mapping, thereby assessing whether a feature consistently predicted higher or lower BMI across CV partitions (32) (Supplementary Methods).

We applied this BMI predictor to the HC_validation_ to assess the model’s generalizability. Furthermore, the BMI predictor was applied to SCZ, ROD and CHR individuals to obtain brain-based BMI predictions for these clinical populations. BMIgap was calculated by subtracting the measured from the predicted BMI scores (BMIgap = BMI_predicted_–BMI_measured_), where a positive BMIgap reflected a higher brain-based prediction of BMI than the measured BMI, and vice versa. To mitigate systematic bias in predicted BMI, characterized by over-estimation at lower BMI ranges and under-estimation at higher BMI ranges, BMIgap were adjusted for BMI through partial correlation analysis (33) (Supplementary Methods, Figure S3-S4). We used the corrected BMIgap for all further analysis steps. Furthermore, we independently calculated BMIgap for antipsychotic and antidepressant naive CHR and ROD individuals to investigate whether medication influenced BMIgap in these subpopulations. Since, there were no unmedicated SCZ patients, we correlated BMIgap with their chlorpromazine equivalents.

### Clinical Investigation of BMIgap

To understand the clinical implications of BMIgap, we analyzed its relationship with clinical variables, particularly within the SCZ group. First, to identify brain patterns distinguishing patients with SCZ from HC individuals, we classified SCZ vs. HC_discovery_ in the MUC sample using the same pre-processing and CV settings as for the BMI-prediction model. The binarized sign-consistency maps derived from SCZ and BMI predictors were overlapped to identify brain regions commonly predictive of both phenotypes (Supplement methods). Furthermore, we extracted the decision scores from the SCZ classifier, which we refer to as SCZ expression score. A higher SCZ expression score indicates a higher likelihood to be classified as SCZ and therefore a greater neuroanatomical similarity to SCZ, while a lower SCZ expression score reflects higher likelihood of HC classification and neuroanatomical HC similarity.

Next, we studied the covariation between BMIgap, SCZ likeness, and clinical variables within the overlapping brain regions of SCZ and BMI. To this end, we employed multivariate SPLS using the SPLS Toolbox by Popovic et al. (34, 35) to investigate covariance patterns between two data domains in the SCZ sample: (i) a six feature matrix including BMIgap, SCZ expression scores, PANSS total score, age of onset, illness duration and number of hospitalizations and, (ii) a brain data matrix containing the vectorized voxels extracted using the binarized mask of overlapping BMI- and SCZ-predictive voxels. The SPLS algorithm uses singular value decomposition to generate multiple layers of distinct, multivariate associative effects between the two data matrices, called latent variables (LV) (Supplementary Methods).

### Investigation of BMIgap and future weight change

We investigated the association between BMIgap and future weight change using two approaches: (i) correlation analysis, and (ii) ML-based prediction of weight gain. In the PRONIA cohort, where longitudinal data was available, we correlated the BMIgap to weight changes at 1- (T1) and 2-year (T2) follow-up. Weight changes were calculated as the difference between the weight at the follow-up time point and the weight at baseline (T0): 1-year (ΔW_1_ = Weight_T1_-Weight_T0_), and 2 years (ΔW_2_ = Weight_T2_-Weight_T0_). Correlation analyses were conducted for the entire cohort, as well as separately for sex and study group. In the first step, we analyzed the correlation between BMIgap and all observed weight changes. Building on previous literature, we correlated BMIgap to weight changes only within subpopulations of patients that exhibited at least + 3%, + 5% or + 7% weight gain at the respective follow-up (36–38). Additionally, we analyzed these correlations across different age ranges, including individuals over 15, 20, 25, 30, and 35 years, as well as within 5-year bins: 15–20, 20–25, 25–30, 30–35, and 35–40 years. Finally, we used the three weight gain thresholds (+ 3%, + 5%, and + 7%) as classification criteria to predict whether CHR and ROD individuals experienced weight gain above these thresholds at T1 and T2 or not. We used BMIgap as well as age, sex, study group (ROD, CHR), exercise (strenuous exercise or mindfulness activities such as yoga and meditation), and history of somatic comorbidities (i.e. whether the individual suffered from somatic illness) as features. Classification analyses were conducted with and without BMIgap as a feature to assess if BMIgap significantly impacts the prediction of future weight gain.

## Results

### Individualized BMI prediction

**Figure 1:**
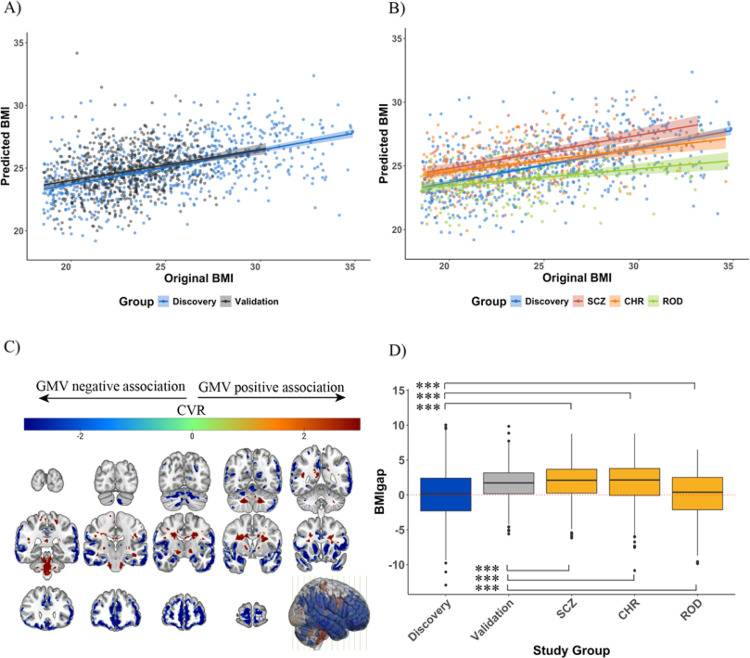
Visualization and performance of the BMI predictor

The model predicted BMI in the HC_discovery_ individuals with an MAE of 2.75 kg/m^2^ (R^2^ = 0.28,P < 0.001) and generalized to the HC_validation_ with an MAE of 2.29 kg/m^2^ for HC (R^2^ = 0.26,P < 0.001, Fig. 1 a). Applied to the clinical subpopulations, the BMI predictor yielded an MAE of 2.85 kg/m^2^ for SCZ (R^2^ = 0.25,P < 0.001), an MAE of 3.07 kg/m^2^ for CHR (R^2^ = 0.16,P < 0.001) and an MAE of 2.73 kg/m^2^ for ROD (R^2^ = 0.10,P < 0.001) individuals (Table 2; Fig. 1 b).

Lower GMV in the cerebellar, prefrontal, occipital, and insular cortices, the postcentral gyrus, hippocampus, and thalamus was predictive of higher BMI. Lower GMV in the left hemisphere involving the cingulate, cerebellar, inferior occipital and temporal cortices, as well as in the right hemisphere covering parts of the precuneus, putamen and Rolandic operculum were predictive of lower BMI (Fig. 1 c, Figure S5, Table S3).

### BMIgap estimation across clinical groups

The application of the BMI predictor yielded a mean (± SD) BMIgap of 0.23 (± 1.68) kg/m^2^ for HC_validation_, 1.05 (± 1.53) kg/m^2^ for SCZ, −0.82 (± 1.64) kg/m^2^ for ROD and 0.51 (± 1.68) kg/m^2^ for CHR individuals. BMIgap differed between HC_discovery_ and HC_validation_ individuals and clinical groups (Fig. 1d; F_[discovery vs. patients]_ = 33.90, P < 0.001; F_[validation vs. patients]_ = 32.36, P < 0.001). Notably, post-hoc pairwise comparisons revealed significant differences in BMIgap between the HC_discovery_ and HC_validation_ groups (t = 2.62, P = 0.009) likely due to variations in BMI distributions, as indicated by a significant difference in variances (F = 3.8018, P<0.001; Figure S1A,C). BMIgap differed significantly between HC_discovery_ and all clinical groups with the highest BMIgap for SCZ (t = 6.68,P < 0.001), followed by CHR (t = 3.72,P< 0.001) and the lowest for ROD (t=−5.88,P < 0.001).

We did not find significant differences in BMIgap between medication naive (N = 80 (37.56%)), antipsychotic-naive (N = 153 (71.83%)), antidepressant-naive (N = 108 (50.70%)) and concurrently antidepressant-antipsychotics-treated (N = 133 (62.44%)) within the CHR individuals (F = 0.6, P = 0.6244). Furthermore, we did not find significant differences between BMIgap of medication naive (N = 59 (29.50%)), antipsychotic-naive (N = 173 (86.50%)), antidepressant-naive (N = 63 (31.50%)) and concurrently antidepressant-antipsychotics-treated (N = 141 (70.50%)) ROD individuals (F = 0.002, P = 0.9964). Moreover, we did not find a significant correlation between BMIgap and chlorpromazine equivalents (r=−0.01,P = 0.86) in the SCZ sample.

### SCZ specific brain-signatures

The SCZ classifier yielded a BAC of 72.4% (sensitivity = 72.2%, specificity = 72.6%; P < 0.001) in separating HC from SCZ individuals. Voxels predictive of SCZ were found predominantly in the inferior, middle and superior frontal gyrus, as well as in hippocampal, thalamic, insular, Rolandic operculum, postcentral, cerebellar, and basal ganglia structures. On the right hemisphere the lingual, fusiform gyrus and mid-temporal lobe were predictive of the SCZ class membership (Figure S6). The brain patterns of the BMI predictor and the SCZ classifier overlapped in the inferior, middle and superior frontal gyrus, caudate, putamen, Rolandic operculum, right precuneus and the middle temporal lobe regions (Figure S7).

### Clinical associations of shared SCZ and BMI signatures

**Figure 2:**
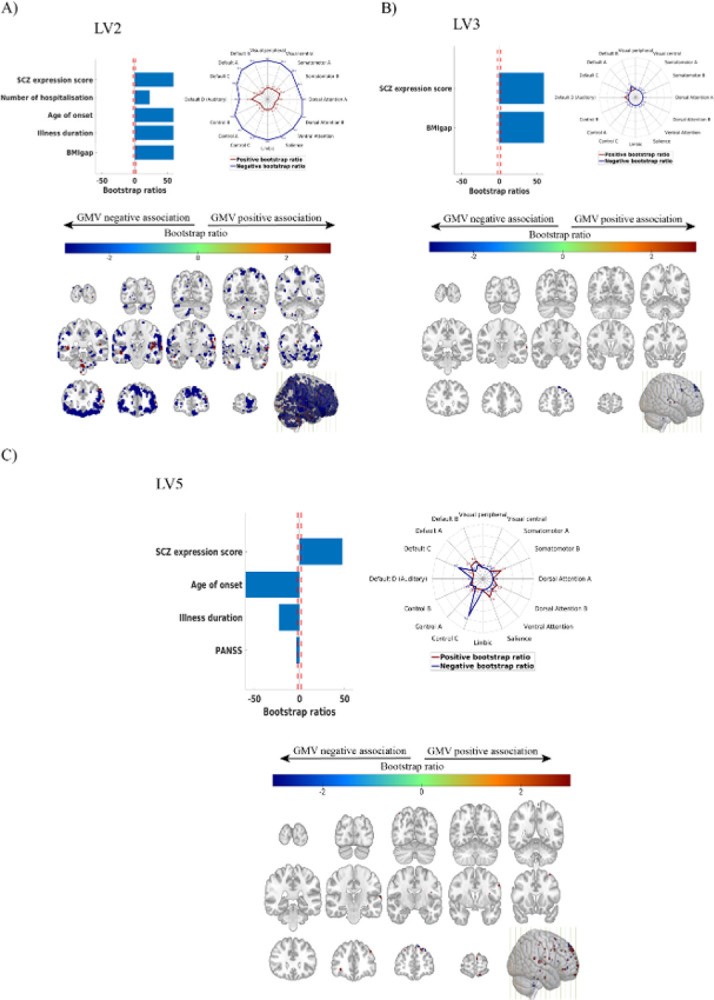
SPLS Analysis of clinical domains and shared SCZ-BMI brain regions (A) LV2, (B) LV3 and (C) a BMIgap-independent signature of LV5.

The SPLS analysis yielded five significant LVs, representing distinct levels of association between the neuroanatomic overlap regions of the BMI and SCZ models and the clinical disease features (Fig. 2, Figure S8). While LV2, LV3, and LV5 captured disease-specific patterns, LV1 and LV4 extracted covariate patterns of age and sex (Supplement Results).

In LV2 (*r* = 0.84, P< 0.001), higher BMIgap, SCZ expression scores, age of onset, number of hospitalizations and illness duration were related to decreased GMV in the default mode network (DMN) specifically in the A, B and C subcomponents, visual, somatomotor-A, attention, salience, limbic, and control networks and increased GMV in DMN (auditory) and somatomotor B networks (Fig. 2a).

In LV3 (*r* = 0.85, P< 0.001), higher BMIgap and higher SCZ expression scores were related to decreased GMV in the DMN-B and increased GMV in the DMN-D (auditory) (Fig. 2b).

In LV5 (*r* = 0.58, P< 0.001), lower PANSS total score, illness duration, and age of onset and higher SCZ expression score were related to decreased GMV in DMN-C and Control-C networks, and increased GMV in the DMN-A/B, Somatomotor-B, Dorsal attention-B, and salience networks (Fig. 2c).

### BMIgap and future weight change

**Figure 3:**
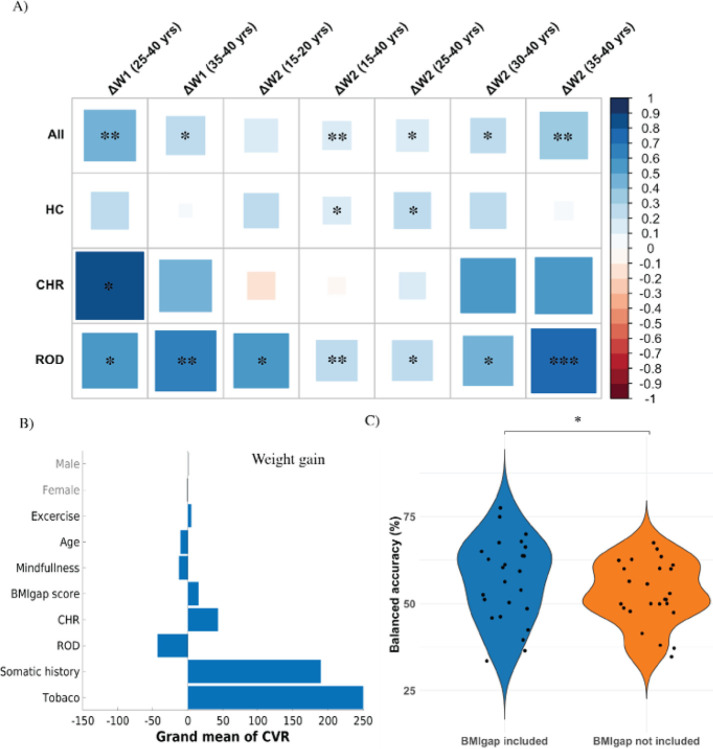
Association between BMIgap and weight change (A) correlation between BMIgap and weight change across different age ranges, time-points and clinical groups, (B) relevant features contributing to predict 7% weight gain, and (C) comparison of model performance between for 7% weight gain at T2 while with and without BMIgap as feature.

In HC individuals, BMIgap showed a positive correlation with 2-year weight gain (ΔW_2_, N = 216; *r* = 0.14,P < 0.05). Specifically, at T2, adults between in the age 25–40 years subgroup (N = 46; *r=* 0.46,P < 0.001). BMIgap of ROD individuals positively correlated with both ΔW_1_ (N = 141; *r=* 0.18,P< 0.05) and ΔW_2_ (N = 92; *r* = 0.3,P< 0.01) at all ages and notably for young individuals between 15–20 years (ΔW_2_:N = 15; *r=* 0.52,P < 0.05). Moreover, female ROD individuals showed a significant correlation between BMIgap and weight gain at T1 (ΔW_1_:N =70; *r=* 0.29,P< 0.05), while male individuals did not (N = 71; r= 0.02,P = 0.86). CHR individuals between 20–40 years of age showed significant correlations between BMIgap and ΔW_1_ in the + 5% weight increase subgroup (N = 58; r= 0.20,P < 0.05) as well as ΔW_2_ in the + 3% (N = 39; r= 0.28,P < 0.05) and + 5% (N = 32; r= 0.36,P < 0.05) subgroup (Fig. 3A, Figure S10).

### Weight gain predictor

The multivariate weight gain prediction model indicated a weight gain of + 7% at T2 with a BAC of 59.2% (sensitivity = 64.9%, specificity = 53.5%, P < 0.05), while the + 3% (BAC = 52.2%; sensitivity = 52.5%, specificity = 51.9%, P = 0.21) and + 5% weight gain prediction models did not perform above chance level (BAC = 45.2%, sensitivity = 62.5%, specificity = 35.2%) (Table S6). Key predictive features for the 7% weight gain predictor included age, clinical group (ROD, CHR), exercise, somatic history, BMI gap, and tobacco use. Specifically, amongst CHR individuals, a higher BMIgap, in combination with a history of somatic conditions and reduced mindfulness-based exercises was predictive of weight gain. For ROD individuals, the BMIgap was negatively associated with 7% weight gain, particularly in individuals with history of lower somatic comorbidities, reduced tobacco use, and higher age (Fig. 3C). Notably, the predictive performance of the + 7% weight reduced after excluding BMIgap as a feature (BAC = 56.1%; sensitivity = 48.6%, specificity = 63.6%; P < 0.05) and these two models differed significantly (P < 0.05) (Fig. 3C).

## Discussion

The aim of our study was to introduce BMIgap as a metric to evaluate BMI-related brain signatures, interrogate its overlaps with SCZ brain patterns and explore its implications for future weight gain.

The BMI predictor relied on lower GMV in frontal and temporal regions and higher GMV in precuneus, putamen and Rolandic operculum to predict BMI. Individuals in the psychosis spectrum (SCZ and CHR) showed an increased BMIgap while individuals suffering from affective diseases (ROD) showed a decreased BMIgap. Moreover, we found that separability of SCZ from HC individuals partly overlapped with BMI-related structural brain variation pertaining to the inhibitory control and reward systems. SPLS analysis revealed that a prefronto-temporal brain pattern predicting both disease and BMI phenotypes was associated with longer illness duration, later disease onset, and higher number of hospitalizations. The potential clinical utility of increased BMIgap, i.e., having a higher brain-estimated BMI than currently measured, was elucidated by our post-hoc analyses. On a group level, higher BMIgap was correlated with future weight gain, an effect particularly pronounced in longer-term trajectories of younger individuals, who were suffering from depressive disorders. On an individual level, BMIgap was identified as a predictive feature for future weight gain in combination with age, clinical group (ROD and CHR), exercise, history of somatic comorbidities, and tobacco usage.

The co-occurrence of higher BMI and lower GMV in the reward and salience systems in the BMI predictor model may represent a neurobiological mediator of eating behaviors (16–19). Furthermore, BMI-predictive GMV reductions in brain areas related to taste, reward, and inhibitory control may contribute to increased susceptibility to hypercaloric eating (17, 19). In patient groups, the SCZ signature and the BMI signatures overlapped within inhibitory, reward and cognitive control regions, thereby pointing to interacting pathways between obesity and SCZ consistent with previous research (20, 39). Our findings substantiate previous reports that prefrontal deficits might lead to reduced cognitive control and therefore may amplify the risk of addictive behaviors such as overeating which could significantly contribute to an increased BMI in psychiatric patients (17, 18, 39).

Positive BMIgap in SCZ and CHR groups indicate that these patients have brain alterations typically found in healthy individuals with higher BMI. This may imply that underlying pathophysiological processes–such as inflammation, insulin resistance, and disruptions in gut microbiota–may influence brain structure similarly to the effects seen in higher BMI conditions and ultimately predispose them to future weight gain (40, 41). These interaction effects may be more pronounced in SCZ compared to CHR individuals given their more advanced disease stage, resulting in higher BMIgap and a higher propensity towards unfavorable metabolic outcomes. Conversely, a negative BMIgap in the ROD group resembles brain signatures found in individuals with a lower BMI. This is supported by previous findings showing overlapping brain patterns between depressed and underweight individuals as well as patients with comorbid depression and anorexia nervosa (42).

Moreover, occurrence of metabolic syndromes in psychiatric patients is often linked to psychiatric medication, namely antipsychotics and antidepressants (11, 43, 44). Antipsychotics impact the mesolimbic dopaminergic system and the ventromedial nucleus, thereby altering behavioral responses to environmental stimuli and regulating both food intake and bodyweight (43). While antidepressants impact metabolic risk by increasing appetite and suppressing satiety, primarily through histaminergic and serotonin receptor antagonism (11, 12). However, we did not find significant differences when comparing the BMIgap of fully naive, partly naive and concurrently antidepressant-antipsychotics-treated CHR or ROD. Moreover, we did not find any significant correlations between BMIgap and antipsychotic dosage in SCZ patients. These findings indicate that the BMIgap captures a neurobiological BMI signature, which may represent a more disease-specific or individual predisposition toward future weight changes. This finding aligns with previous research indicating that patients with psychiatric disorders, are at an increased risk of developing metabolic syndrome and obesity, independent of medication use (45, 46), contrasting other literature that primarily attributes weight gain to the effects of psychiatric medication (11, 12).

The phenotypic association analysis linked control and reward brain networks to SCZ diagnosis, BMIgap and clinical variables in unique ways. The multivariate signatures of concurrently high BMIgap and SCZ expression scores (LV2, LV3) were associated with both a decrease in GMV within the limbic network and increased GMV within the DMN (auditory) network. In obesity research, these networks have been involved in reward processing (47), food motivation (48), executive and affective control (45), while in SCZ research, they have been particularly implicated in the impaired processing of negative emotions (49). Furthermore, later disease onset, higher hospitalization frequency, and longer illness duration, were associated with both higher BMIgap and SCZ-expression scores, thereby implying a potential association between the severity of SCZ and the presence of obesity risk traits. Moreover, we identified a pattern independent of BMIgap (LV5) suggesting that there are distinct effects of SCZ and BMI on the brain, indicating that those with early onset, shorter illness duration, and milder symptoms are less likely to show high SCZ-diagnostic separability, thus, highlighting the complexity of SCZ subtypes and the variability in its manifestation and progression.

Moreover, our assertion that the BMIgap is associated with future weight changes was supported by the positive correlation between BMIgap and future weight change observed across all groups, with the strength of this correlation increasing from 1 to 2-year follow-up. Notably, these correlations were most pronounced among young individuals (15–20 years) in the ROD group and young adults (25–40 years) in the CHR group, indicating that individuals in the early stages of psychiatric illness may exhibit distinct brain signatures that predispose them to future weight gain. Furthermore, the 7% weight gain predictor, highlighted that ROD young adults who have higher exercise rates, use less tobacco, have less somatic comorbidities, and a lower BMIgap are less likely to experience weight gain. Conversely, younger CHR individuals with reduced exercise rates, more history of somatic comorbidities, higher tobacco use, and a higher BMIgap were at an increased risk for future weight gain. Notably, the exclusion of the BMIgap feature from the 7% weight gain predictor resulted in a significant reduction of predictive accuracy of the model in correctly predicting future weight gain. Therefore, the BMIgap may be helpful in identifying patients at risk of weight gain, facilitating the early implementation of tailored interventions such as weight-neutral medications, individualized dietary and physical activity regimens, to stabilize weight (24, 25).

### Limitations

Among the limitations of our study is the lack of individuals with very high (> 35) or low (< 18) BMI, which should be further addressed by future studies focusing on the understudied subpopulations of underweight and highly obese individuals. Moreover, the study lacked metabolic markers necessary to further interrogate the shared pathophysiological substrates affecting brain structure and clinical covariations between obesity and psychiatric disorders. However, BMI, being widely used in similar studies, enabled us to make preliminary and direct comparisons with prior findings (16–18, 39, 50).

## Conclusion

In conclusion, our study identified BMIgap as a crucial metric for exploring the relationship between BMI and brain structure in psychiatric populations, particularly within the depression and schizophrenia spectrum. We found that elevated BMIgap was associated with significant neurobiological alterations in reward and inhibitory control systems, indicating a complex interplay between obesity and schizophrenia. Our findings further suggest that BMIgap could potentially be a predictive indicator of future weight gain, especially among younger individuals with a higher disease burden. Therefore, BMIgap could serve as a template of using machine learning and brain imaging to enhance early identification of patients at risk for metabolic complications. Integrating BMIgap, or future, more sophisticated tools, into clinical assessments may improve strategies for preventing weight gain in psychiatric patients.

## Figures and Tables

**Table 1:** Sociodemographic differences in discovery, validation, and clinical groups.

	HC		t/χ^2^	P	Clinical group			F/χ^2^	P
	Discovery	Validation			SCZ	CHR	ROD		
Sample (N)	770	734	0.86^b^	0.35	146	213	200	776.60^b^	**< .001**
BMI, Mean (SD)	25.10 (4.03)	23.03 (2.07)	12.49^a^	**< .001**	24.02 (3.40)	23.46 (3.42)	24.01 (3.57)	13.48^c^	**< .001**
Age, Mean (SD)	41.26 (15.51)	32.24 (12.75)	12.29^a^	**< .001**	30.83 (9.97)	23.92 (5.24)	26.02 (6.37)	164.42^c^	**< .001**
Sex (Female, N (%))	435 (56.49)	373 (50.82)	4.75^b^	**0.029**	34.00 (23.29)	103 (48.36)	96 (48.00)	55.17^b^	**< .001**
Symptoms, Mean (SD)									
PANSS (total)	NA	NA	NA	NA	52.26 (29.45)	52.07 (18.22)	48.44 (14.26)	2.08^c^	0.13
PANSS (positive)	NA	NA	NA	NA	11.92 (8.13)	11.31 (4.16)	8.20 (2.21)	29.09^c^	**< .001**
PANSS (negative)	NA	NA	NA	NA	14.98 (9.73)	13.09 (6.86)	12.50 (5.66)	_5_^c^	**.007**
PANSS (general)	NA	NA	NA	NA	25.36 (16.13)	27.66 (10.05)	27.74 (8.84)	2.19^c^	.113
SANS (total)	NA	NA	NA	NA	44.44 (26.52)	27.08 (24.88)	23.91 (20.95)	30.69^c^	**< .001**
BDI	3.55 (5.05)	3.27 (5.20)	0.50^a^	0.616	NA	24.09 (11.75)	24.56 (12.48)	182.47^c^	**< .001**
Functioning, Mean (SD)									
GAF:S past month	86.30 (6.43)	87.32 (6.16)	1.54^a^	0.124	NA	51.31 (11.29)	53.92 (13.17)	503.97^c^	**< .001**
GAF:D past month	85.49 (6.32)	86.51 (5.89)	1.59^a^	0.113	NA	51.94 (12.)	54.14 (3.60)	385.24^c^	**< .001**
GF:S current	8.50 (0.89)	8.54 (0.71)	0.51^a^	0.612	NA	6.25 (1.75)	6.39 (1.23)	157.82^c^	**< .001**
GF:R current	8.49 (0.77)	8.59 (0.67)	1.40^a^	0.164	NA	5.80 (1.70)	5.97 (1.81)	150.54^c^	**< .001**
WHOQoL (total)	97.45 (33.60)	102.81 (28.91)	1.65^a^	0.099	NA	71.20 (28.81)	74.92 (24.86)	39.58^c^	**< .001**
Medications									
Antipsychotics (N (%))	NA	NA	NA	NA	NA	60 (28.17)	27 (13.50)	12.51^b^	**<0.001**
Antidepressants (N (%))	NA	NA	NA	NA	NA	105 (49.30)	137 (68.50)	4.23^b^	**0.040**
CPZ-equivalent (mg)	NA	NA	NA	NA	358.9 (382.4)	NA	NA	NA	NA

a χ^2^-test,

b t-test,

c F-test

**Table 2:** Model performances of the regression analysis for the discovery model and its application to the replication and patient groups.

Study group	N	BMIgap uncorrected (kg/m^2^)	BMIgap (kg/m^2^)	MAE (kg/m^2^)	R^2^	*r*	P
HC_discovery_	770	−0.01 (3.4)	0 (1.78)	2.75	0.28	0.53	**< 0.001**
HC_validation_	734	1.73 (2.2)	0.23 (1.68)	2.29	0.26	0.51	**< 0.001**
SCZ	146	1.83 (3.0)	1.05 (1.53)	2.85	0.25	0.50	**< 0.001**
CHR	213	1.70 (3.26)	0.51 (1.68)	3.07	0.16	0.40	**< 0.001**
ROD	200	−0.03 (3.48)	−0.82 (1.64)	2.73	0.10	0.32	**0.001**
